# Central Pain Due to Injury of the Spinothalamic Tract Misdiagnosed as Complex Regional Pain Syndrome: A Case Report

**DOI:** 10.3390/diagnostics9040145

**Published:** 2019-10-08

**Authors:** Sung Ho Jang, Young Hyeon Kwon, Sung Jun Lee

**Affiliations:** Department of Physical Medicine and Rehabilitation, College of Medicine, Yeungnam University 317-1, Daemyungdong, Namku, Daegu 705-717, Korea; rehab6467@hanmail.net (S.H.J.); kyh7648764@daum.net (Y.H.K.)

**Keywords:** diffusion tensor tractography, spinothalamic tract, traumatic axonal injury, whiplash injury, complex regional pain syndrome

## Abstract

Objectives: We report on a patient with whiplash injury who had central pain, due to injury of the spinothalamic tract (STT), but who was misdiagnosed as complex regional pain syndrome (CRPS). Case description: While a minivan in which a 43-year-old female was seated in the passenger seat was stopped for a signal, a truck collided with the minivan from behind, and the minivan then repeatedly collided with trucks in front and behind the minivan. Her head repeatedly struck the minivan seat resulting in whiplash injuries. After onset, she felt pain in both legs with mild motor weakness in all four extremities and memory impairment. Eight years after onset, she was diagnosed at a university hospital as CRPS type 1 with the clinical features of hyperalgesia and mild edema and motor weakness of both legs. She visited another university hospital nine years after onset and complained of pain in the right arm and both legs, constant tingling and burning pain along with allodynia and hyperalgesia. She also showed mild weakness in the four extremities, mild edema of both legs, and memory impairment. On diffusion tensor tractography (DTT), the left spinothalamic tract (STT) showed marked narrowing, and the right STT revealed mild narrowing and partial tearing. In addition, partial tears were observed in both corticospinal tracts and the right corticoreticulospinal tract. Discontinuations were observed in the left corticoreticulospinal tract and the left fornical crus. Conclusion: Injury of the STT was demonstrated on DTT in a patient with central pain following whiplash injury. Previously, the patient was misdiagnosed as CRPS.

## 1. Introduction

Complex regional pain syndrome (CRPS) is a debilitating condition that affects the limbs and can be induced by surgery or trauma [1]. Brain injury is also a precipitating factor in CRPS type 1 [2,3]. Precise diagnosis of CRPS is important because a misdiagnosis can complicate recovery and impair a patient’s functional state [1]. Moreover, a misdiagnosis of CRPS is often possible because there is no gold standard approach to the diagnosis of this condition. Regardless, the diagnostic criteria for CRPS are based on clinical features [2,4,5,6].

Whiplash is a bony or soft tissue injury resulting from an acceleration-deceleration energy transfer to the neck [7]. Patients with whiplash injury often complain of cerebral symptoms suggestive of brain injury, and previous studies have reported signs indicative of brain injury following whiplash injury by using functional neuroimaging, voxel-based morphometry, and brain nuclear medicine applications [8,9]. However, a clear demonstration of neural injury has been limited. Recently, with the introduction of diffusion tensor imaging (DTI), several studies have used diffusion tensor tractography (DTT), which is derived from DTI data, to demonstrate traumatic axonal injury (TAI). In patients with whiplash injury, TAI is the tearing of axons, due to indirect shearing forces during acceleration, deceleration, and rotation of the brain [10,11,12,13,14,15,16,17,18].

In this case report, we report on a patient with whiplash injury who showed central pain, due to injury of the spinothalamic tract (STT) that was initially misdiagnosed as CRPS.

## 2. Case Report

**Figure 1:**
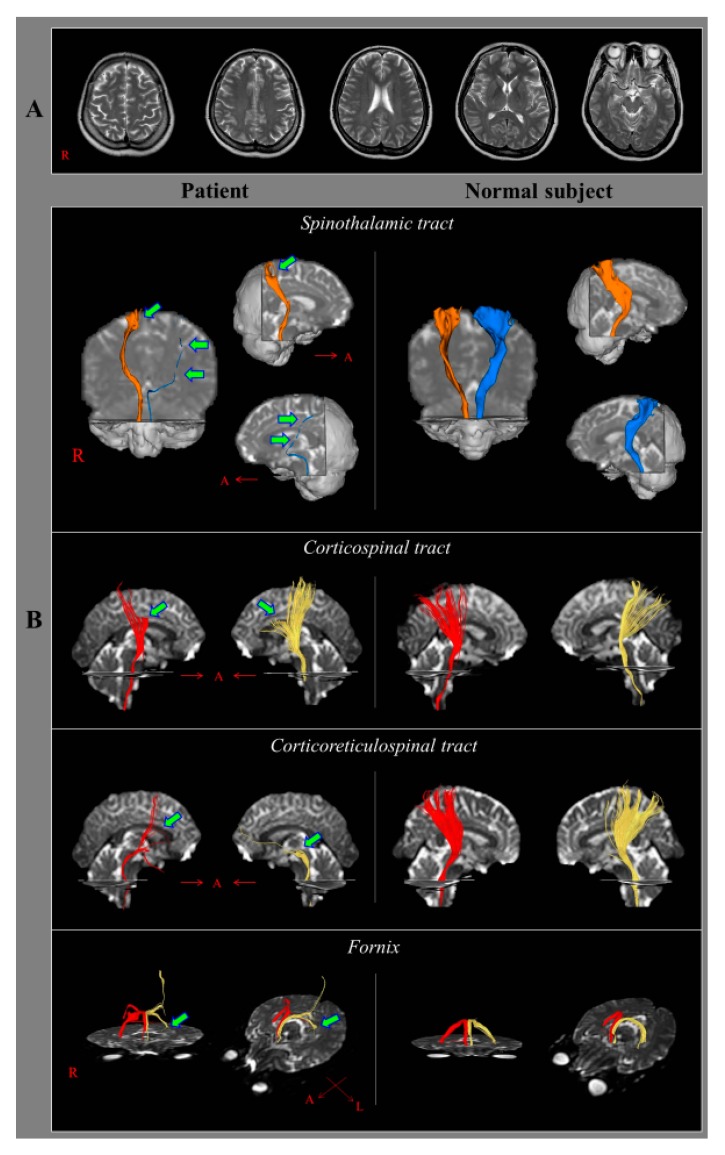
(**A**) T2-weighted brain magnetic resonance images at nine years after whiplash injury onset shows no abnormality. (**B**) Results of diffusion tensor tractography of the patient at nine years after injury compared to those from a normal subject (42-year-old female). The left spinothalamic tract shows marked narrowing (green arrows) while the right spinothalamic tract reveals mild narrowing and partial tearing in the upper portion (green arrow). In addition, partial tears at the subcortical white matter (green arrows) are visible in both corticospinal tracts and the right corticoreticulospinal tract. Discontinuations (green arrows) are observed in the left corticoreticulospinal tract and the left fornical crus. Orange color—right spinothalamic tract; blue color—left spinothalamic tract; red color—right corticospinal tract, corticoreticulospinal tract, fornix, yellow color—left corticospinal tract, corticoreticulospinal tract, fornix; A: anterior, R: right, L: left.

A 43-year-old female patient suffered from head trauma resulting from a car accident. She was seated in the passenger seat of a minivan was stopped at a signal light behind a truck, and another truck collided with her car from behind. The minivan then repeatedly collided with the trucks in front and behind. During that collision, her head repeatedly struck the minivan seat resulting in whiplash injuries. The patient reported that she did not experience loss of consciousness or post-traumatic amnesia. The patient’s initial Glasgow Coma Scale score was 15. After the head trauma, she felt pain in both legs, mild motor weakness in all four extremities, and memory impairment. Although she visited several hospitals to determine the cause of her pain, she was unable to obtain a precise diagnosis for her pain because the conventional brain and whole spine MRIs did not show any abnormality. At eight years from onset, she was diagnosed as CRPS type 1 with the clinical features of hyperalgesia and mild edema and motor weakness of both legs; moreover, abnormality was not detected on plain radiography for hand and leg, three-phase bone scan, and thermography. Nine years after the head trauma, she visited the rehabilitation department of our university hospital complaining of pain in the right arm and both legs. The characteristics and severity of the pain were as follows: Constant tingling and burning sensation with allodynia and hyperalgesia (visual analog scale score: Right arm and leg, 9; left leg, 7) [19]. She also exhibited mild weakness of the four extremities (4^−^/4), mild edema of both legs, and memory impairment. However, trophic changes of the arm and legs, including skin and nails, were not observed. In addition, she mentioned that she did not experience the distal edemas on the right arm and both legs, which could observe during the acute stage of CRPS [20]. Electromyography for all extremities and trunk failed to detect any abnormality (Figure 1A). The patient provided signed, informed consent, and the study protocol was approved by the institutional review board of our university hospital (YUMC 2019-06-032, approved on 21 June 2019).

## 3. Diffusion Tensor Imaging

DTI data were acquired nine years after onset by using a sensitivity-encoding head coil on a 1.5 T Philips Gyroscan Intera (Hoffman-LaRoche, Best, Netherlands). For each of the 32 non-collinear diffusion sensitizing gradients, 70 contiguous slices were acquired parallel to the anterior commissure-posterior commissure line. Imaging parameters were as follows: Acquisition matrix = 96 × 96; reconstructed matrix = 192 × 192; field of view = 240 mm × 240 mm; TR = 10,726 ms; TE = 76 ms; parallel imaging reduction factor (SENSE factor) = 2; EPI factor = 49; b = 1000 s/mm^2^; NEX = 1; and 2.5 mm slice thickness with no gap. For assessment of the STT, the Oxford Centre for Functional Magnetic Resonance Imaging of the Brain (FMRIB) Software Library (www.fmrib.ox.ac.uk/fsl) was used to analyze the obtained DTI data. Affine multi-scale two-dimensional registration was used for correction of head motion effect and image distortion, due to eddy currents. Fiber tracking was performed by using a probabilistic tractography method based on a multi-fiber model and was applied in the current study by utilizing tractography routines implemented in FMRIB Diffusion software (5000 streamline samples, 0.5 mm step lengths, curvature threshold = 0.2). To examine the STT, the seed region of interest (ROI) was placed on the posterolateral medulla on an axial slice, and target ROIs were placed on the portion of the ventro-postero-lateral nucleus of the thalamus and the primary somatosensory cortex [15,21]. The threshold of two streamlines was applied to the results of fiber tracking for each tract. To reconstruct the corticospinal tract (CST) and corticoreticulospinal tract (CRT), fiber tracking was performed by using the fiber assignment continuous tracking algorithm implemented within DTI task card software (Philips Extended MR Work Space 2.6; Philips, Amsterdam, Netherlands) [15]. Each DTI replication was intra-registered to the baseline ‘b0’ images to correct for residual eddy-current image distortions and head motion effects by using a diffusion registration package (Philips Medical Systems; Philips, Amsterdam, Netherlands). For analysis of the CST, the seed ROI was placed on the anterior blue portion of the upper pons on the axial image of the color map, and the target ROI was placed on the anterior blue portion of the lower pons on the axial image of the color map. Regarding the reconstruction of the CRT, the seed ROI was placed on the reticular formation of the medulla, and the target ROI was placed on the tegmentum of the midbrain. Fiber tracking of the CST and CRT was performed by using a fractional anisotropy (FA) threshold of >0.15 and a direction threshold of <27°. In addition, for the reconstruction of the fornix, the seed ROI was placed at the junction between the body and column of the fornix, and target ROIs were placed on each side of the crus of the fornix [22]. Fiber tracking of fornix was performed by using an FA threshold of >0.2 and a direction threshold of <45°.

The DTT results for the patient showed that the left STT had marked narrowing, while the right STT had mild narrowing and partial tearing in the upper portion. In addition, partial tears at the subcortical white matter were observed in both CSTs and the right CRT; discontinuations were observed in the left CRT and the left fornical crus (Figure 1B).

## 4. Discussion

In this case report, by using DTT, we were able to detect injuries of the STT, CST, CRT, and fornix in a patient with whiplash injury, and the neural injuries were consistent with the clinical features of this patient; that is, central pain (injury of the STT), quadriparesis (injury of the CST and CRT), and memory impairment (injury of the fornix) [10,11,12,13,14,15,16,17,18]. Because no definite brain lesion was observed on conventional brain MRI, TAI appeared to be the most likely pathogenetic mechanism for injuries of these neural tracts [18,23]. As a result, we concluded that the patient’s previous diagnosis of CRPS type 1 was incorrect for the following reasons: (1) She was diagnosed based on the clinical features of hyperalgesia with mild edema and motor weakness of both legs without additional supportive evidence from plain radiography of hand and leg, three-phase bone scan, and thermography. We consider the hyperalgesia to be ascribable to the STT injury, and the mild edema and motor weakness of the legs are ascribable to the CST and CRT injuries as those tracts are involved in leg motor function; (2) [10,12,15]. The patient did not have a history of the distal edema, which is a characteristic feature of acute stage CRPS, and she failed to show tropic changes of skin and nail at the chronic CRPS stage [20]. In contrast, the patient showed other symptoms of brain injury, such as memory impairment which is not a symptom of CRPS.

To date, using DTT, three methods for the diagnosis of TAI of a neural tract in patients with mild traumatic brain injury, including whiplash injury have been suggested: (1) Configurational analysis of a DTT-reconstructed neural tract (abnormal findings: Partial tearing, narrowing, discontinuation, and non-reconstruction); (2) measurement of DTI parameters using ROIs that are applied on the partial injury site of a neural tract on DTT; and (3) statistical comparison of DTT parameters of a neural tract of an individual patient with those for age-, sex-, and handedness-matched control subjects [10,11,12,13,14,15,16,17,18].

In conclusion, injury of the STT was demonstrated in a patient with central pain following a whiplash injury. Based on our results, the patient was misdiagnosed as CRPS type 1. Therefore, we suggest that analysis of the STT by performing DTT is useful in the diagnosis of TAI, particularly in patients who complain central pain following whiplash injury.
